# Effect of melatonin on male offspring testis and sperm parameters in BALB/c mice after exposing their mother to METHamphetamine during pregnancy and lactation

**DOI:** 10.22038/IJBMS.2023.69608.15158

**Published:** 2023

**Authors:** Fatemeh Ghorbani, Sareh Karimi, Arad Boustan, Alireza Ebrahimzadeh-bideskan, Ehsan Saburi

**Affiliations:** 1 Department of Anatomy and Cell Biology, Faculty of Medicine, Mashhad University of Medical Sciences, Mashhad, Iran; 2 Department of Medical Biotechnology and Nanotechnology, Faculty of Medicine, Mashhad University of Medical Sciences, Mashhad, Iran; 3 Applied Biomedical Research Center, Faculty of Medicine, Mashhad University of Medical Sciences, Mashhad, Iran; 4 Medical Genetics and Molecular Medicine Department, Faculty of Medicine, Mashhad University of Medical Sciences, Mashhad, Iran

**Keywords:** Lactation, Melatonin, Methamphetamine, Pregnancy, Testis

## Abstract

**Objective(s):**

Methamphetamine (METH) is a psychostimulant that has harmful effects on all organs, the nervous system, cardiovascular system, and reproductive system. Since many METH consumers are young people of reproductive age, it poses a risk to the next generation of METH consumers. METH can pass through the placenta and is also secreted into breast milk. Melatonin (MLT) is the primary hormone of the pineal gland that regulates the circadian cycle, and it is also an antioxidant that can mitigate the effects of toxic substances. This study aims to investigate the protective effect of melatonin against the detrimental effects that METH has on the reproductive system of male newborns, whose mothers consumed METH during pregnancy and lactation.

**Materials and Methods:**

In the current study, 30 female adult balb/c mice were divided into three groups: control group, vehicle group that received normal saline, and the experimental group that received 5 mg/kg METH intraperitoneally during gestation and lactation. After lactation, the male offspring of each group were randomly divided into two subgroups, one of which received 10 mg/kg melatonin intragastrically for 21 days (corresponding to the lactation period of the mice) (METH-MLT) and the other did not (METH -D.W). After treatment, the mice were sacrificed and testicular tissue and epididymis were obtained for the following tests.

**Results:**

The diameter of seminiferous tubules, SOD activity, total Thiol groups concentration, catalase activity, sperm count, and PCNA and CCND gene expression were significantly increased in the METH-MLT group compared with the METH-DW. Apoptotic cells and MDA level ameliorated in the METH-MLT group compared with METH-D.W, and testicular weight had no notable change.

**Conclusion:**

The current study represents that consumption of METH during pregnancy and lactation can have adverse effects on the histological and biochemical factors of testis and sperm parameters of male newborns, which can be mitigated by taking melatonin after the end of the breastfeeding period.

## Introduction

Methamphetamine (METH) is a central nervous system stimulant that distributes more rapidly in the CNS than amphetamine, making it more pleasurable to drug users (1). The primary forms of METH are powder and crystal (2). The mortality of METH abuse is due to its cardiovascular effects (1). It also has an adverse impact on other organs such as the immune, nervous, digestive, and reproductive systems (1, 3). Since most users of METH are adolescents and adults of reproductive age, METH is an important factor in reproductive toxicology (4, 5). METH has different harmful effects on the male reproductive system, such as an increase in apoptotic cells in seminiferous tubules and a decrease in sperm count and normal morphology (6, 7). METH consumption causes excessive production of reactive oxygen species (ROS) and suppresses the antioxidant enzyme system, so this imbalance causes oxidative stress (8). METH also can affect the cell cycle and inhibit cell proliferation by interfering with the expression of some genes, such as PCNA and CCND (9). Due to the low molecular weight and high-fat solubility of METH, it can pass the blood-brain barrier and the placenta and is also found in breast milk (10, 11). It has been reported that newborns of mothers who consume METH usually suffer adverse physical and behavioral consequences (12). 

Melatonin (MLT) is a hormone that is mainly produced by the pineal gland in the brain. It is a regulator of the circadian cycle which regulates the sleep-wake rhythm (13). It also has antioxidant and anti-inflammatory properties that scavenge free radicals(14). MLT influences the male reproductive system and regulates testicular development (15).

As there is insufficient evidence on the effects of METH usage during pregnancy and lactation on the reproductive system of male newborns and the curative effect of MLT after METH usage, in the current study, we will investigate the efficacy of consumption of MLT for mitigating the negative effects of METH abuse during pregnancy and lactation on the reproductive system of male newborns.

## Materials and methods

In this study, 30 female and 15 male adult BALB/c mice were provided by the animal care center of Mashhad University of Medical Sciences. The animals were kept in an air-conditioned animal care center at 23±2 ^°^C, relative humidity of 55±5%, and a 12-hour light-dark cycle. The animals had free access to standard pellet feed and water.

Every two female mice were housed with one male mouse for mating at 5:00 PM. The following day, the female mice were checked, and the presence of a vaginal plug was determined to signify pregnancy day zero (0). Twenty-four pregnant female mice were randomly divided into three groups, including 1) the control group (Ctrl) without any intervention, 2) the saline (Sal) group that received saline, and 3) the METH group that received METH (5 mg/kg). METH was dissolved in normal saline and injected daily by intraperitoneal (IP) injection during pregnancy and lactation (16). After lactation, male offspring at the age of 3 weeks, were randomly divided into two subgroups: A) received MLT at a dose of 10 mg/kg just before the dark and B) received distilled water (D.W) by gavage (17) for 21 days. 

After the treatment period, the animals were sacrificed to remove their testes and epididymis, left testis was fixed in 10% normaline (normal saline+formaldehyde) for histological study, and the right testis for testis weight, oxidative stress evaluation, and real-time PCR assessment.

***Testes weight***

The right testes were weighed by a 0.1 mg precise digital scale.

***Biochemical measurements***

For biochemical measurements, testis tissues were homogenized. For this purpose, 1 ml of cold phosphate-buffered saline (PBS) was added to 100 mg of testes tissue, and the tissue was then homogenized by a mechanical homogenizer.

*Malondialdehyde (MDA)*

MDA level is an index of lipid peroxidation. A solution containing 375 mg of TBA, 15 mg of TCA, and 2 ml of HCl was prepared, and the total volume was made up to 100 ml. One milliliter of homogenized testis tissue was added to 2 ml of complex solution, and put in a boiling water bath for 45 min. Then it was placed at room temperature and after cooling it was centrifuged at 1000 rpm for 10 min, the supernatant was removed, and its absorbance was read at 535 nm(18, 19). The concentration of MDA was calculated using the following formula:

C(M)=Absorbance ⁄1.65×10^5^

*Superoxide dismutase (SOD)*

SOD activity was measured according to the procedure described by Madesh and Balasubramanian method. A colorimetric assay involving the production of superoxide through autoxidation of pyrogallol and inhibiting the reduction of MTT (3-(4, 5-dimethylthiazol-2-yl) 2, 5-diphenyltetrazolium bromide) to formazan was performed. 

An appropriate amount of homogenized tissue, MTT, and pyrogallol was added to the plate and incubated in a dark place at room temperature. DMSO was added and shaken gently to inhibit the reaction. Finally, the absorbance was read at 570 nm using a microplate reader (Epoch Biotek, USA). One unit of SOD activity was defined as the amount of enzyme that caused 50% inhibition of the MTT reduction rate (18).

*Measurement of total thiol groups (SH)*

From the reaction of the DTNB reagent with SH groups, a yellow complex is created, which is used to measure thiol groups. This complex has an absorption peak at the wavelength of 412 nm with an optical absorption coefficient of 13.6 mM^-1^cm^-1^. A mixture of 1 ml of Tris-EDTA buffer (pH=8.6) and 50 μl of tissue homogenate was prepared and its absorbance was read at 412 nm against a blank (A1). Then, 20 μl of DTNB reagent was added to this mixture, and after 10 min of incubation at room temperature, absorbance was read again (A2). The absorbance of the DTNB reagent was read as blank (B). The total thiol concentration (mM) was calculated according to the following formula (20):

Total thiol concentration (mM)=(A_2_-A_1_-B)×1.07/0.05×13.6

*Catalase (CAT) assay*

The activity of the CAT enzyme was measured by the Aebi method as a function of the ability to degrade H_2_O_2_. Using the slope of the H_2_O_2_ absorption curve, CAT enzyme activity was calculated and normalized to protein concentration (21).

***Histological methods***

*Seminiferous tubule diameter*

Testicular tissue was prepared for H&E staining (hematoxylin and eosin) according to standard histological methods. After fixation in normalin for two days at room temperature, specimens were dehydrated with ascending graded alcohol, cleared with xylene, and then embedded in paraffin. The paraffin blocks were cut into 5 µm thickness by a rotatory Leitz microtome, and finally, the tissue sections were stained with H&E.

To measure the seminiferous tubule diameter, five photographs were randomly taken from each slide using ×40 objective lens of a light microscope (BX51, Olympus, Japan) equipped with a high-resolution camera (DP10). Each image was analyzed using the image processing software analySIS LS Starter. Tubule diameter was measured in the cross sections of seminiferous tubules (22).

*TUNEL method*

The terminal deoxynucleotidyl transferase dUTP nick end labeling (TUNEL) method was used to detect apoptotic cells using the TUNEL kit (Roche, Germany). 

Two sections of each specimen were placed on the slides coated with Poly-L-lysine. After deparaffinization and rehydration, the internal peroxidase was neutralized with 3% H_2_O_2_, and after washing, 15 μl of protein kinase was added for 20 min, after washing again, the enzyme and enzyme label were added and incubated for 24 hr in the refrigerator. After washing again, 15 μl of POD (horseradish peroxidase) was add, one hour of incubation was done, then 15 μl of DAB was added to the sections and then stained with hematoxylin. In this method, apoptotic cells are detected by dark brown nuclei. Tissue sections were evaluated and photographed using ×40 objective lens of a light microscope (Olympus BX 51, Japan). The number of TUNEL-positive cells was counted and calculated according to the following formula (23):

NA=ΣQaf×ΣP

“NA” is the number of apoptotic cells per area, “ ∑Q”, “a/f”, and “ ∑P” represent the sum of counted apoptotic cells, the area associated with each frame, and the sum of frame-associated points hitting the defined space, respectively.

***Sperm parameters***

To evaluate sperm parameters, epididymis was cut into five pieces and placed in 1 ml of normal saline, and incubated at 37 ^°^C for 15 min. Then, sperm count, sperm morphology, and DNA fragmentation index were measured using the following techniques.

*Sperm count*

To count sperm, 10 μl of the solution was added to a Neubauer slide after incubation, and sperm were measured in 25 squares using a light microscope (Olympus BX51) at 40x_ magnification, and the average amount of sperm counted was reported. The number of sperm per milliliter was calculated using the following formula:

$n=\frac{\mathrm{mean of sperms in four square}\times \mathrm{delutin}(1)}{\mathrm{volume of one square}(100\mathrm{nl})}$

*Sperm morphology*

Papanicolaou staining was performed to study sperm morphology. For this purpose, 50 μl of sperm solution was smeared on a glass slide and fixed with methanol (70%), then the slide was stained. On each slide, 200 sperms were counted under a light microscope (40x magnification). The sperms without tails, with curled or bent tails, or with two or abnormal heads were recognized as abnormal sperms.

*DNA Fragmentation Index (DFI)*

Sperm DNA fragmentation was assessed using the SDFA kit (Ideh Varzan Farda Co. Tehran, Iran) according to the manufacturer’s instructions. The sperm count reached 5-10×10^6 ^/ml, 50 ul of sperm solution was mixed with melted agarose, and 30 µl of the cell suspension was placed on a coated slide glass (included in the kit) and covered with a coverslip and incubated at 4 ^°^C. After 5 min, the coverslip was carefully removed, and the denaturing solution was added and incubated for 7 min at room temperature. Then the slides were immersed in a lysis solution. After 15 min, the glass slides were washed with distilled water. The glass slides were dehydrated with alcohol and then stained using the kit protocol. 

After covering the slide with a coverslip, the spermatozoa were observed under a light microscope at 40 magnification. DNA fragmentation was classified based on spermatozoa halo size. Spermatozoa with a large or medium halo were scored as having no fragmentation (normal spermatozoa), and spermatozoa with a small or no halo were scored as indicating fragmentation (DNA-fragmented spermatozoa). A total of 200 spermatozoa were examined and scored in each sample. The fragmentation rate was calculated as DFI (%)=(fragmented spermatozoa/total number of spermatozoa counted)×100 (24).

***Quantitative real-time PCR***

The total RNA of testicular tissue was isolated using the Total RNA Extraction Kit (Parstous Co. Tehran, Iran) according to the manufacturer’s protocol. Purification and concentration of the extracted RNAs were measured using NanoDrop 2000C (Thermo Scientific, USA). One microliter of the extracted RNA was used for cDNA synthesis based on the manufacturer’s instructions (Paratous Co. Tehran, Iran) (25). Quantitative real-time PCR was performed using specific primers for proliferating cell nuclear antigen (PCNA) and CCND1 and GAPDH (as housekeeping gene), which are listed in Table 1. QRT-PCR reactions were conducted on the Light cycler96 system (Roche, Switzerland) using SYBR Green master mix (Parstous Co. Tehran, Iran). All experiments were duplicated, and the relative fold-change of genes was calculated using the 2^-^^ΔΔCt^ method. Standard curves for the target (PCNA, CCND1) and reference (GAPDH) genes were generated (26).

***Statistical analysis***

Statistical analysis was performed using Prism v.8.4.3 software (GraphPad Software). Data were analyzed using one-way ANOVA and Tukey’s *post hoc*. Data were expressed as mean±SD, and *P*-values less than 0.05 were considered statistically significant.

## Results

***Testes weight***

**Figure 1:**
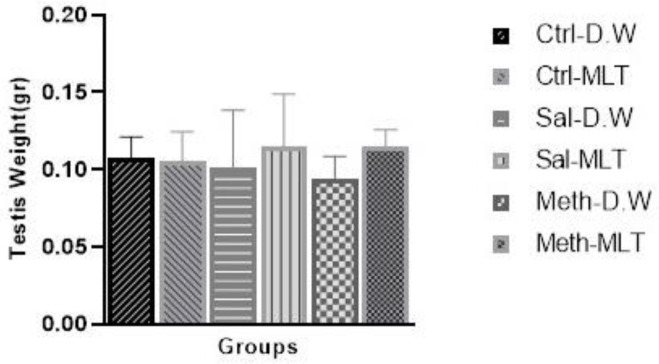
Comparison of the weight of the testicles in the rats examined in the study based on different groupings

There was no significant difference in testis weight between the studied groups (Figure 1).

***Seminiferous tubule diameter***

**Figure 2:**
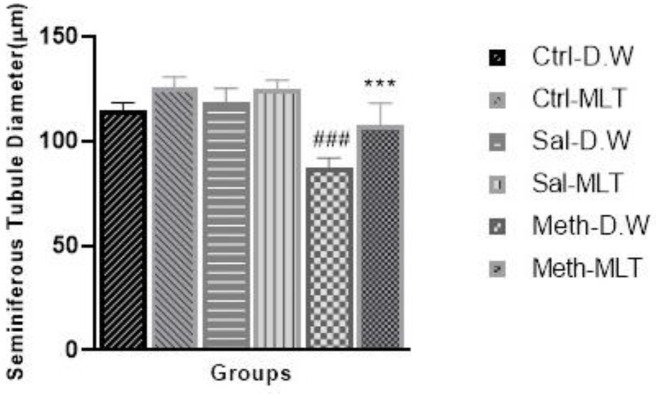
Comparison of seminiferous tubule diameter in testis sections stained with Hematoxylin and Eosin (H&E) staining

^###^ indicates *P≤*0.001 in comparison with Ctrl-D.W, *** indicates *P≤*0.001 in comparison with METH-D.W, METH-MLT had no significant difference with Ctrl-DW, Mean±SEM

The diameter of seminiferous tubules was significantly decreased in the METH-D.W group compared with the other control and saline groups and improved in the METH-MLT group compared with the METH-D.W group (*P*≤0.001). The METH-MLT group had no significant difference compared with the Ctrl-D.W and Sal-D.W groups (Figure 2).

***Biochemical measurements***

*MDA*

MDA level in the METH-D.W group was significantly increased compared with Ctrl-D.W and Ctrl-MLT and Sal. D.W, and Sal-MLT groups (*P*≤0.01), and also compared with METH-MLT (*P*≤0.05), and there were no significant differences between other groups. 

*SOD*

**Figure 3:**
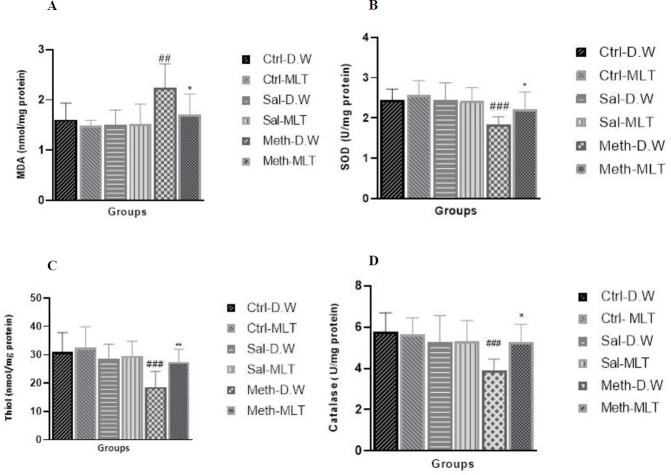
Comparison of MDA level (A), SOD activity (B), Thiol concentration (C), and catalase enzyme activity (D) between groups. ^###^ indicates *P≤*0.001 in comparison with Ctrl-D.W, ^##^ indicates *P≤*0.01 in comparison with Ctrl-D.W, ** indicates *P≤*0.01 in comparison with METH-D.W, * indicates *P≤*0.05 in comparison with METH-D.W, Mean±SEM

The SOD activity in the METH-D.W group decreased compared with METH-MLT (*P*≤0.05), and also in comparison with Ctrl-D.W and Ctrl-MLT, and Sal. D.W and Sal-MLT groups (*P*≤0.001), but no significant differences were observed between other groups (Figure 3A).

*Thiol groups*

Thiol concentration significantly decreased in the METH-D.W group compared with Ctrl-D.W and Ctrl-MLT and Sal. D.W and Sal-MLT groups (*P*≤0.001) and METH-MLT (*P*≤0.01). There was no significant difference between the other groups (Figure 3C). 

*CAT assay*

The CAT activity decreased in the METH-D.W group compared with Ctrl-D.W and Ctrl-MLT groups (*P*≤0.001), and Sal. D.W and Sal-MLT groups (*P*≤0.01). The CAT activity in the METH-MLT group was higher than in the METH-D.W group (*P*≤0.05) (Figure 3D).

***TUNEL assay***

**Figure 4:**
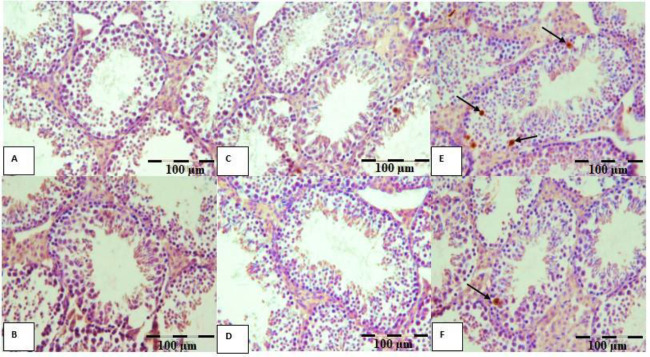
Photomicrographs of testicular tissue sections show the TUNEL positive cells, the arrows represent TUNEL positive cells (dark cells)

**Figure 5:**
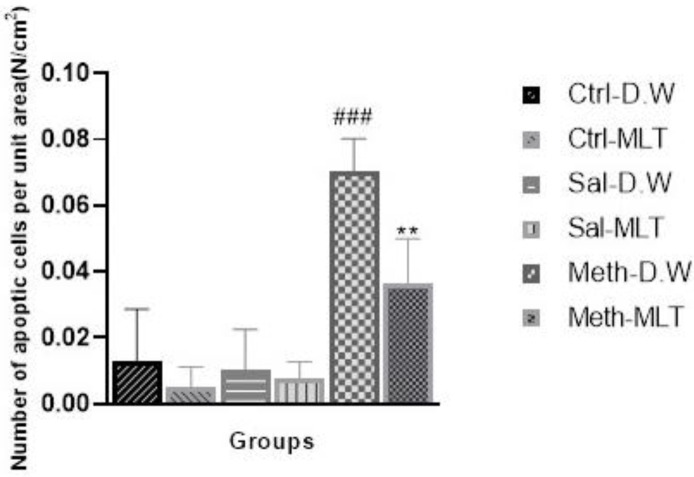
Comparison of apoptotic cells in studied groups

^#^^##^ indicates *P≤*0.001 in comparison with Ctrl-D.W, ** indicates *P≤*0.01 in comparison with METH-D.W, the difference between METH-MLT and Ctrl-D.W was not significant, Mean±SEM

The number of apoptotic cells was increased in the METH-D.W group compared with all other groups (*P*≤0.001), and in METH-MLT group was lower than in METH-D.W group (*P*≤0.01), (Figures 4 and 5). 

***Sperm parameters***

*Sperm count*

**Figure 6:**
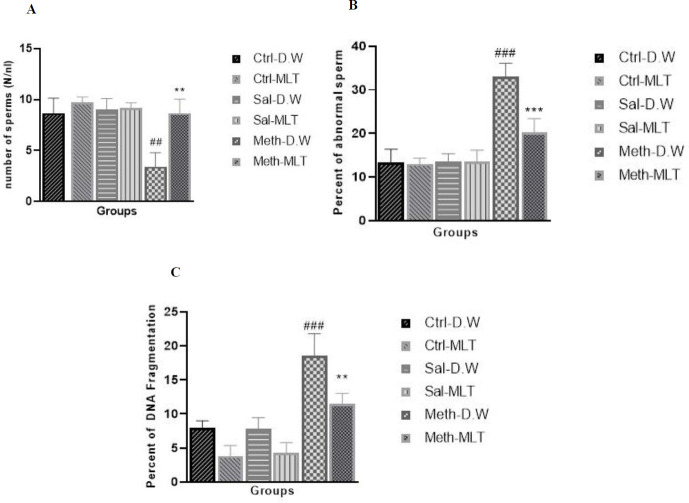
Comparison of sperm parameters, sperm count (A), sperm morphology (B), and DFI (C)) in studied groups

^###^ indicates *P≤*0.001 in comparison with Ctrl-D.W, *** indicates *P≤*0.001 in comparison with METH-D.W, ^##^ indicates *P≤*0.01 in comparison with Ctrl-D.W, ** indicates *P≤*0.01 in comparison with METH-D.W, Mean±SEM

The results of sperm count show that this parameter significantly decreased after METH exposure in the METH-D.W group compared with the other groups (*P*≤0.001). Sperm count was significantly increased in the METH-MLT group compared with the METH-D.W group (*P*≤0.01, Figure 6A). 

*Sperm morphology*

Morphology of sperms was investigated using Papanicolaou staining and the results showed** t**hat the percentage of abnormal sperms increased in the METH-D.W group compared with other groups. At the same time, it decreased in the METH-MLT group compared with the METH-D.W group (*P*≤0.001, Figure 6B).

***DNA fragmentation index (DFI)***

The percentage of damaged DNA was significantly increased in the METH-D.W group compared with all other groups (*P*≤0.001). This parameter was decreased in the METH-MLT group compared with the METH-D.W group (*P*≤0.01). The METH-MLT group had no significant difference in comparison with Ctrl-D.W and Sal-D.W, but it was significantly decreased compared with Ctrl-MLT and Sal-MLT groups (*P*≤0.001), (Figure 6C).

***Real-Time PCR***

*PCNA*

**Figure 7:**
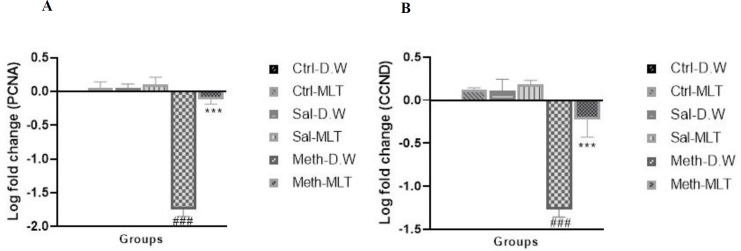
Comparison of the Log fold change of PCNA (A) and CCND (B) expression

^###^ indicates *P≤*0.001 in comparison with Ctrl-D.W, *** indicates *P≤*0.001 in comparison with METH-D.W, The reduction observed between METH-MLT compared with Ctrl-D.W was not significant, Mean±SEM

The results of real-time PCR in Figure 7A indicated that the expression of the PCNA gene significantly decreased in the METH-D.W group in comparison with other groups (*P*≤0.001) and increased in METH-MLT compared with METH-D.W (*P*≤0.001). There was no significant difference in the expression of this gene in the METH-MLT group compared with the Ctrl-D.W group (Figure 7A). 

*CCND*

The expression of CCND decreased in the METH-D.W group in comparison with Ctrl-D.W, Ctrl-MLT, Sal-D.W, and Sal-MLT groups (*P*≤0.001), and the expression of this gene was significantly increased in the METH-MLT group compared with the METH-D.W group (*P*≤0.001), but there was no significant difference in comparison with Ctrl-D.W group (Figure 7B).

## Discussion

METH is a psychoactive drug that spreads rapidly in the central nervous system and causes euphoria, increased alertness, increased heart rate, and decreased appetite. The use of METH is increasing among young people who are of reproductive age, and it also exposes a person to risky sexual behaviors (1). The lipophilic nature of METH causes it to pass through the umbilical cord and also to be secreted in breast milk, which causes complications in the fetus, and its use during pregnancy causes problems such as fetal death and premature birth (10).

MLT is a hormone secreted from the pineal gland and one of its functions is to regulate the sleep/wake cycle. In addition, its antioxidant and anti-inflammatory role has also been proven, and it also has an antioxidant and protective role in the male reproductive system (27).

In past studies, the effects of METH on the male reproductive system have been investigated, based on the research conducted, little information is available on the effects of this drug on the reproductive system of male children of mothers using METH during pregnancy and breastfeeding, as well as possible treatment. It is not recommended for these unwanted effects created in the individual.

Free radicals are produced in the body under normal conditions, and they cause destructive effects in the body, including the destruction of proteins, nucleic acids, and cell membranes. To counter these effects, the antioxidant system works and removes or neutralizes free radicals. If the cell is in a situation where the production of free radicals exceeds the normal limit and the antioxidant system is not able to remove them, oxidative stress occurs. Exposure to certain substances and radiation can cause oxidative stress, METH is one of the substances that increase the production of free radicals. ROS produced as a result of the use of METH initiates lipid peroxidation, lipid peroxidation is a chain reaction that is followed by the release of MDA, the high level of MDA in the tissue indicates oxidative stress conditions (28).

 The enzymatic and non-enzymatic antioxidant system prevents the formation of free radicals and can reduce the intensity of oxidative stress. Enzymatic antioxidants such as SOD and CAT play a role in neutralizing superoxide and hydrogen peroxide radicals, which in the condition of imbalance between the antioxidant system and the amount of free radicals produced, the amount of these enzymes in the tissue decreases, which indicates the dominance of free radicals affects this system and causes oxidative stress. Thiol groups are non-enzymatic antioxidants that can directly detoxify ROS and prevent lipid peroxidation. In the case of oxidative stress in the tissue, the concentration of thiols in the tissue decreases (29).

Substances that have antioxidant properties such as MLT, vitamin E, vitamin C, iron, and other known antioxidants can directly affect the antioxidant system and improve its efficiency in neutralizing and eliminating free radicals. The use of these substances increases the amount of antioxidant enzymes SOD and CAT in the tissue, as a result of which the free radicals of superoxide and hydrogen peroxide are neutralized and the amount of stress created in the tissue is reduced; also, the concentration of thiols increases after the use of antioxidants, as a result of which the amount of lipid peroxidation decreases (30).

In the present study, the oxidative effect of METH as well as the antioxidative effect of MLT in the next generation was confirmed by examining the biochemical parameters of MDA, SOD, CAT, and thiol groups. The amount of antioxidant factors decreased due to receiving METH and the amount of MDA increased, on the other hand, after receiving MLT, the enzymatic and non-enzymatic antioxidants in the tissue increased and the amount of MDA in the tissue decreased.

The increase of intracellular ROS, the deprivation of cells from growth factors, and the breakdown of cell DNA cause the initiation of the mitochondrial pathway of apoptosis, the permeability of mitochondria increases and pro-apoptotic proteins are released into the cytosol. Diablohomolog is one of the pro-apoptotic proteins that counteract the cytosolic inhibitors of pro-apoptotic proteins and activate caspase and promote apoptosis. Activated caspases 8 and 9 cause the release of caspases 3, 6, 7, and proteases that direct cell destruction by breaking down/cleaving many proteins and activating DNase. The interaction between proapoptotic and antiapoptotic members of the BCL2 family controls the mitochondrial pathway of apoptosis, BAK and BAX, which are members of this family, are necessary to increase mitochondrial membrane permeability and release cytochrome C, which activates caspase 9. BCL2, BCL-XL, BCLW, MCL1, A1, and BOO/DIVA proteins are essential for cell survival and function. Imbalance in the anti-apoptotic and pro-apoptotic members of the BCl2 family causes the activation of the mitochondrial pathway of apoptosis. METH by increasing the expression of BAX, which is one of the pro-apoptotic proteins of this family, causes an imbalance in these proteins and induces apoptosis (29).

MLT prevents the induction of apoptosis by activating the anti-apoptotic proteins of the Bcl-2 family and reducing the activity of pro-apoptotic proteins such as Bax (31). In this study, the amount of apoptosis induced in the testicular tissue under the conditions of MLT consumption after exposure to METH showed that MLT has an effective role in reducing the amount of induced apoptosis.

Damage to the DNA molecule causes mutagenesis, free radicals attack the DNA molecule and cause it to break and destroy the integrity of this molecule, although the repair of this molecule is a continuous process if the production of radicals increases freely, the rate of DNA breakage exceeds its repair capacity. ROS in the male reproductive system also damages the DNA of the sperm and disrupts its integrity. METH use increases ROS. Its high amount leads to oxidative stress and damage to nuclear and mitochondrial DNA, telomere shortening, and makes it epigenetic (32); the lack of sperm DNA integrity is one of the causes of sperm inefficiency, which affects fertility (33).

The use of METH in the reproductive system of the user causes side effects such as a decrease in the sex hormone testosterone, a decrease in the number and quality of sperm, a decrease in spermatogenesis, and damage to the testicle tissue, which lead to a disruption in the reproductive system. METH reduces spermatogenesis in the male reproductive system by reducing cell proliferation and increasing apoptosis of germ cells, which leads to a decrease in the number of sperms. METH dose-dependently decreases the expression of progesterone and testosterone receptors in spermatogonia and Sertoli cells, and also affects sperm morphology and reduces the percentage of sperm with normal morphology, METH decreases sperm motility, which ultimately leads to a decrease in fertility (3, 34)

The mammalian testis has many unsaturated fatty acids that are susceptible to ROS attack. MLT is a lipophilic and hydrophilic compound that easily passes through the cell membrane, the antioxidant property of MLT protects these fatty acids from ROS attack. MLT consumption reduces caspase activity and also reduces sperm DNA damage caused by H_2_O_2_ activity, both of which are dependent on the expression of MLT-1 receptor and extracellular kinases. Due to the presence of MLT receptors in the hypothalamus and pituitary, we can point to the regulatory role of this hormone in the release of gonadotropin-releasing hormones, FSH and LH in these tissues. Also, MLT receptors in the male reproductive system act as an antioxidant, reduce testicular damage, regulate testicular growth, and as a male fertility protector in mammals. It also protects the morphology and function of sperm by preventing the production of free radicals beyond normal, and it also plays an effective role in spermatogenesis, the abnormal amount of serum MLT leads to male infertility (15). The results of the present study showed the antioxidant and protective effect of MLT in testicular tissue against the destructive effects caused by METH, which are in line with the results reported in previous studies.

Many genes are involved in the regulation of the cell cycle, the decrease and increase of their expression affect cell proliferation. Cyclins are a family of proteins that regulate the cell cycle. One of these proteins is CyclinD1, which is encoded by the CCND1 gene (35, 36). PCNA is also essential for multiple pathways of the cell cycle and plays a role in DNA replication. In the absence of P53 (tumor suppressor) and high amounts of PCNA in the cell, replication occurs. PCNA also interacts with P21 (cell cycle inhibitor)(37). METH increases the expression of P53 and P21, and since DNA replication occurs in the absence of P53, METH thus disrupts DNA replication and reduces cell proliferation, as well as by increasing P21, which inhibits the cell cycle, and thus reduces cell proliferation (38). The data obtained from this study also indicate a decrease in the expression of PCNA and CCND1 genes due to exposure to METH.

MLT, which stimulates cell proliferation in non-cancerous conditions, can increase PCNA gene expression and induce cell proliferation by expressing this gene (39, 40). This feature of MLT was also observed in the present study and the animals exposed to METH and the expression of PCNA and CCND1 genes in them was reduced, it improved significantly with the use of MLT and the expression of these genes in the METH-MLT group increased compared with the METH-D.W group.

**Table 1:** The sequence and temperature characteristics of the primers used in qPCR assay

**Gene**	**Forward primer**	**TM**	**Reverse primer**	**TM**
m-PCNA	AGATGTGCCCCTTGTTGTAGAG	60.03	GAAAAGACCTCAGGACACGC	58.85
m-CCND1	GCGTACCCTGACACCAATCTC	60.74	CTCCTCTTCGCACTTCTGCTC	60.74
m-GAPDH	GGGGTCCCAGCTTAGGTTC	59.39	CCCAATACGGCCAAATCCG	58.97

No significant differences were observed

Ctrl-D.W; control group received distilled water after lactation; Ctrl-MLT; control group received melatonin after lactation; Sal-D.W; saline group received distilled water after lactation; Sal-MLT; saline group received melatonin after lactation; Meth-D.W; methamphetamine group received distilled water after lactation; Meth-MLT; methamphetamine group received melatonin after lactation

Ctrl-D.W; control group received distilled water after lactation; Ctrl-MLT; control group received melatonin after lactation; Sal-D.W; saline group received distilled water after lactation; Sal-MLT; saline group received melatonin after lactation; Meth-D.W; methamphetamine group received distilled water after lactation; Meth-MLT; methamphetamine group received melatonin after lactation

Ctrl-D.W; control group received distilled water after lactation; Ctrl-MLT; control group received melatonin after lactation; Sal-D.W; saline group received distilled water after lactation; Sal-MLT; saline group received melatonin after lactation; Meth-D.W; methamphetamine group received distilled water after lactation; Meth-MLT; methamphetamine group received melatonin after lactation

A=Ctrl-D.W group, B=Ctrl-MLT group, C=Sal-D.W group, D=Sal-MLT group, E=METH-D.W group, F=METH-MLT group

Ctrl-D.W; control group received distilled water after lactation; Ctrl-MLT; control group received melatonin after lactation; Sal-D.W; saline group received distilled water after lactation; Sal-MLT; saline group received melatonin after lactation; Meth-D.W; methamphetamine group received distilled water after lactation; Meth-MLT; methamphetamine group received melatonin after lactation

Ctrl-D.W; control group received distilled water after lactation; Ctrl-MLT; control group received melatonin after lactation; Sal-D.W; saline group received distilled water after lactation; Sal-MLT; saline group received melatonin after lactation; Meth-D.W; methamphetamine group received distilled water after lactation; Meth-MLT; methamphetamine group received melatonin after lactation

Ctrl-D.W; control group received distilled water after lactation; Ctrl-MLT; control group received melatonin after lactation; Sal-D.W; saline group received distilled water after lactation; Sal-MLT; saline group received melatonin after lactation; Meth-D.W; methamphetamine group received distilled water after lactation; Meth-MLT; methamphetamine group received melatonin after lactation

Ctrl-D.W; control group received distilled water after lactation; Ctrl-MLT; control group received melatonin after lactation; Sal-D.W; saline group received distilled water after lactation; Sal-MLT; saline group received melatonin after lactation; Meth-D.W; methamphetamine group received distilled water after lactation; Meth-MLT; methamphetamine group received melatonin after lactation

## Conclusion

MLT can ameliorate the destructive effects of METH on the male reproductive system by neutralizing ROS, so, taking MLT as a protective agent is recommended. This finding needs further investigation because if we cannot reduce METH abuse, we can try to reduce side effects in newborns of METH-consuming mothers.

## Authors’ contributions

FG, ES, AEB, SK, and AB were involved in the search strategy and drafting. All authors supervised all steps of the project, revised and edited the manuscript, and read and approved the final manuscript.

## Funding

The authors would like to appreciate the Research Deputy of Mashhad University of Medical Sciences for supporting this study (IR.MUMS.REC. 1398.778).

## Ethics approval and consent to participate

Not applicable.

## Consent for publication

Consent for publication is given by all authors. 

## Conflicts of interest

The authors declare that they have no competing interests.
